# Sero-epidemiological study on Dengue fever virus in humans and camels at Upper Egypt

**DOI:** 10.14202/vetworld.2020.2618-2624

**Published:** 2020-12-10

**Authors:** Mostafa Osman Hussen, Amal S. M. Sayed, Mostafa F. N. Abushahba

**Affiliations:** 1 Tahta Veterinary Hospital, Tahta, Sohag Governorate, Egypt; 2 Department of Zoonoses, Faculty of Veterinary Medicine, Assiut University, Asyut 71526, Egypt

**Keywords:** camel, dengue virus, Egypt, prevalence, zoonosis

## Abstract

**Background and Aim:**

Dengue fever (DF) is an important mosquito-borne viral zoonosis affecting over 100 countries worldwide and putting about 3.9 billion people at risk of infection. The disease has re-emerged in Egypt since 2011; however, there is a paucity of recent epidemiological data available. Therefore, in this study, we employed a cross-sectional study to determine DF prevalence in humans and camels in Asyut and Sohag Governorates, Egypt, during 2019.

**Materials and Methods:**

A total of 91 humans and a similar number of dromedary camels were utilized in this study. Sera were obtained and analyzed for the presence of specific antibodies against DF virus using enzyme-linked immunosorbent assay. Related epidemiological data affecting the disease spread in humans and camels were recorded and statistically analyzed.

**Results:**

The seroprevalence of DF in humans and camels was 12.09% and 3.3%, respectively. The disease varied significantly by the species examined as humans were found to be at a higher risk of acquiring the infection compared to camels. Nearly equal odds of exposure (odds ratio [OR]) were seen in the individuals with close contact with camels compared to those without; however, individuals exposed to mosquitoes were at approximately 3 times higher risk of infection (OR=2.95 [95% confidence interval [CI], 0.73-11.93]) compared to individuals who were not exposed to mosquitoes (OR=0.033 [95% CI, 0.084-1.37]). Interestingly, DF seropositivity in camels was significantly related to the presence or absence of symptoms within 2 weeks before sampling (p=0.02) where symptomatic animals had higher odds of exposure (OR=19.51 [95%, 0.97-392.3]) compared to asymptomatic ones (OR=0.05 [95%, 0.002-1.03]).

**Conclusion:**

The current study reports the presence of specific antibodies against dengue virus (DENV) in humans residing within Asyut and Sohag Governorates, Egypt. Furthermore, it provides the first serological evidence of DENV circulation in camels which is alarming. A more comprehensive study is needed; however, this baseline investigation underscores the urgent need for increasing awareness among people residing in the area as well as application of the appropriate mosquito control measures to avoid further spread of the disease.

## Introduction

Dengue fever (DF) is a mosquito-borne zoonotic disease that affects humans and non-human primates [1]. The disease is caused by the dengue virus (DENV) which is known to have four distinct serotypes (DENV-1-4) and belongs to the family Flaviviridae, which also includes chikungunya, yellow fever, and Zika viruses. Each of these viruses is known to be transmitted by the female mosquitoes mainly of the species *Aedes aegypti* and, to a lesser extent, *A. albopictus*, which become vectors for viral transmission after taking a blood meal from an infected host [1]. DF has dramatically expanded its endemicity pattern from localized epidemics affecting only nine countries before 1970 to the current pandemic in which the virus is circulating in 129 countries, putting an estimated 3.9 billion people across the globe at risk of infection [2].

Following an incubation period usually ranging from 4 to 10 days [3], dengue infection can vary from a subclinical infection without any detectable symptoms in 20-80% of cases to a more pronounced flu-like disease [4]. While illness can be mild in some individuals, infection results in severe symptoms and pathology in up to half a million people worldwide every year. This is marked by the manifestation of severe bleeding, organ impairment, and/or plasma leakage [5]. Neither specific treatments nor approved vaccines are currently available for dengue infection, leaving the entire global population at risk of infection in any upcoming pandemic. Compounding this problem further is evidence indicating that the generation of antibodies to any of the four serotypes not only fails to protect against the other strains but also increases the risk of developing severe dengue on subsequent exposure to any of the other DENV serotypes [6-9]. However, the fact that this phenomenon can occur in any individuals who have been exposed to the virus, including individuals who remained asymptomatic [7], represents a vexing challenge which warrants the need for continuous monitoring of the individuals in the endemic areas along with the application of the necessary mosquito control and public health education programs. The first dengue outbreak in Egypt was documented as early as 1799, namely, in Cairo and Alexandria Governorates [10]. Several outbreaks have occurred subsequently in different areas of Egypt through the year 1937 at which 2594 human cases were reported [11]. Since 1940, a decline in the disease incidence has been noted following the introduction of dichlorodiphenyltrichloroethane and the subsequent reduction of *Aedes* populations [12]. In 2010, concerns about the re-emergence of *Aedes* mosquitoes in Egypt had been raised when two Italian tourists were diagnosed with dengue infection following visiting South Egypt [13]. Five years later, an outbreak occurred in humans residing in Dariot district, Asyut Governorate; however, it was rapidly contained after application of the appropriate control measures [14]. Recently, in 2017, up to 2500 human cases of DF disease were reported in the Red Sea Governorate [15] where *A. aegypti* was documented to be circulating [16].

Although there are some limited reports of the recent uptick in disease prevalence in South Egypt and its significant implications on public health, the epidemiological situation of the disease in Egyptians and the contribution of animals in its cycle are understudied. Therefore, we employed this preliminary cross-sectional study to determine the prevalence and the associated risk factors of DF in humans and camels in two South Egyptian governorates, namely, Asyut and Sohag.

## Materials and Methods

### Ethical approval and informed consent

Verbal informed consent was taken from all participants, and they were informed that the information obtained would remain strictly confidential. Parenteral consent was taken before the participation of individuals <18 years old. Animal experiments were carried out following the regulatory rules of Assiut University for animal research and samples were obtained after the agreement of owners. The method of consent and both the animal and human work were approved by the Ethics Committee of Assiut University, Egypt.

### Study area, duration, and sampling criteria

The current survey was conducted from January 2019 to May 2019 and included two different governorates in Upper Egypt, namely, Sohag (26°33′N 31°42′E) and Asyut (27.252°N 31.09°E). A sum of 91 human sera was recruited for this study, including 57 males and 34 females. All feverish individuals of the known cause were excluded. Data including residence, gender, age, contact with camels, exposure to mosquitoes, and presence of breakbone fever at or within 14 days prior to sampling were obtained with in-person interviews.

A total of 91 blood samples were randomly collected from camels within the two investigated areas, including Sohag (79) and Asyut (12). All the sampled camels were adults (76 males and 15 females) primarily imported for slaughtering purposes with an age of 6 years and above. For each sample, camel’s sampling site, age, gender, breed, breeding purpose, and any history of fever within the 14 days before sampling were recorded.

### Serum samples

Blood samples (5 CC) were collected directly into plain Vacutainer tubes from humans or restrained camels through venipuncture. The samples were transferred immediately to the laboratory at the Department of Zoonoses, Assiut University, where sera were separated through centrifugation at 1500× *g* for 15 min. Sera were then stored at −20°C for later processing [17].

### Serology

Sandwich anti-DENV immunoglobulin G (IgG) human and camel ELISAs (Sinogeneclon Co., Ltd., China) were used in the present study to detect the anti-DENV-specific IgG antibodies in human and camel sera, respectively. Briefly, a volume of 10 μl of serum from each sample was used for the assay and the test procedure was then completed according to the manufacturer’s protocol. The color change was measured spectrophotometrically, 15 min after stopping the reaction, at a wavelength equal to 450 nm using a BioTek Model Elx800 Absorbance Microplate Reader (Fisher Scientific, Lenexa, KS, USA). The cutoff of each assay was obtained as follows: Cutoff=The average optical density of negative control + 0.15. Samples with an OD ≥ the calculated cutoff were considered positive and samples which had OD values < he calculated cutoff were judged as negative.

### Statistical analysis

Categorical variables were analyzed by Fisher’s exact test and odds ratios (OR) with 95% confidence interval (CI) in the GraphPad Prism 6.0 software (GraphPad Software, Inc., La Jolla, CA, USA). p<0.05 was considered statistically significant.

## Results

In this study, as shown in Table-1, a seroprevalence of 12.9% and 3.30% of DENV was detected in humans and camels, respectively. Seroprevalence was significantly different based on the species examined (p=0.048) and humans were at more than 4 times greater risk (OR=4.03 [95% CI, 1.09-14.98]) of getting the infection compared to camels (OR=0.25 [95% CI, 0.07-0.92]).

**Table-1:** Seroprevalence of dengue virus in humans and dromedary camels.

Species examined	No. examined	Positive no. (%)	Negative no. (%)	Odds ratio (95% CI)
Human	91	11 (12.09)	80 (87.91)	4.03 (1.09-14.98)
Camel	91	3 (3.30)	88 (96.70)	0.25 (0.07-0.92)
Total	182	14 (7.69)	168 (92.31)	p=0.048

CI=Confidence interval

As revealed in Table-2, none of the analyzed variables had a significant effect on the seropositivity of the sampled humans; however, the OR revealed differences in the exposure rate between the included subcategories. Individuals exposed to mosquitoes had higher odds of exposure (OR=2.95 [95% CI, 0.73-11.93]) compared to those that did not (OR=0.033 [95% CI, 0.084-1.37]). The age group ranging from 1 to 20 years was at the highest risk of getting the infection (OR=3 [95% CI, 0.41-22.09]) compared to the other two groups (21-40 years and 40 years and above). Breakbone fever, the prominent sign of DENV, was seen in 81.81% of the seropositive individuals, while the remaining seropositive individuals were asymptomatic.

**Table-2:** Impact of different factors on dengue virus seropositivity in humans.

Variable	No. examined	Positive no. (%)	Negative no. (%)	Odds ratio (95% CI)
Residence				
Rural	69	9 (13.04)	60 (86.96)	1.5 (0.30-7.53)
Urban	22	2 (9.09)	20 (90.91)	0.67 (0.13-3.35) p=1.00
Gender				
Male	57	8 (14.03)	49 (85.96)	1.69 (0.41-6.85)
Female	34	3 (8.82)	31 (91.18)	0.59 (0.15-2.41)p=0.53
Age				
<21 years	8	2 (25)	6 (75)	3 (0.41-22.09)
21-40 years	53	6 (11.32)	47 (88.68)	1.15 (0.26-4.97)
>40 years	30	3 (10)	27 (90)	Reference p=0.49
Contact with camels				
Yes	25	3 (12)	22 (88)	0.99 (0.24-4.07)
No	66	8 (12.1)	58 (87.90)	1.01 (0.25-4.16) p=1.000
Exposure to mosquitoes				
Yes	46	8 (17.39)	38 (82.61)	2.95 (0.73-11.93)
No	45	3 (6.67)	42 (93.33)	0.033 (0.084-1.37) p=0.197
Presence of breakbone fever				
Yes	70	9 (12.85)	61 (87.14)	1.4 (0.29-7.06)
No	21	2 (9.52)	19 (90.48)	0.71 (0.14-3.59)p=1.00

CI=Confidence interval

As depicted in Table-3, the seropositivity in camels did not differ significantly in accordance with sampling locality, gender, age, breed, or breeding purpose. Although an insignificant association between such factors and the seropositivity was found in this study, the OR did reveal discrepancies between the subcategories. This discrepancy was apparent in both gender and breed of the sampled camels, as female camels (OR=2.64 [95 CI, 0.22-31.19]) and imported breed (OR=3.03 [95 CI, 0.26-34.70]) had higher odds of exposure compared to male camels (OR=0.378 [95 CI, 0.03-4.46]) and local breeds (OR=0.33 [95 CI, 0.03-3.78]), respectively. Moreover, a slight difference in the OR was detected between the subcategories of breeding purpose, sampling locality, and age. Interestingly, all three of the seropositive camels were obtained from Sohag and had fever and/or respiratory symptoms at the time of sampling.

**Table-3:** Impact of different factors on dengue virus seropositivity in camels.

Variable	No. examined	Positive no. (%)	Negative no. (%)	Odds ratio (95% CI)
Sampling locality				
Asyut	12	0 (0.00)	12 (100)	0.87 (0.04-17.98)
Sohag	79	3 (3.80)	76 (96.20)	1.14 (0.056-23.52)p=1.000
Gender				
Male	76	2 (2.63)	74 (97.37)	0.378 (0.03-4.46)
Female	15	1 (6.67)	14 (93.33)	2.64 (0.22-31.19) p=0.42
Age				
6-9 years	29	1 (3.45)	28 (96.55)	1.07 (0.09-12.32)
≥10 years and above	62	2 (3.23)	60 (96.77)	0.93 (0.08-10.74) p=1.000
Breed				
Local	54	1 (1.85)	53 (98.15)	0.33 (0.03-3.78)
Imported	37	2 (5.40)	35 (94.60)	3.03 (0.26-34.70) p=0.56
Breeding purpose				
Work	48	2 (4.17)	46 (95.83)	1.83 (0.16-20.89)
Slaughtering	43	1 (2.33)	42 (97.67)	0.55 (0.048-6.26) p=1.000
Symptoms				
Presence of “fever/loss of appetite”	26	3 (11.54)	23 (88.46)	19.51 (0.97-392.3)
Absence of “fever/loss of appetite”	65	0 (0.00)	65 (100)	0.05 (0.002-1.03)p=0.02

CI=Confidence interval

## Discussion

During the past 5 years, two outbreaks of DF have affected people in Upper Egypt, namely, Dairot District, Asyut Governorate (2015) and El Quseir, Red Sea Governorate (2017) where the mosquito vector, *Aedes* was found to be prevalent. Yet, to the best of our knowledge, there are currently no available epidemiological data regarding the disease in the area to date. Relevant studies are particularly important in defining new areas possibly affected and the possible role of animals in the disease cycle. Therefore, in this study, we determined the prevalence of DENV in humans and camels, as well as the associated risk factors in Upper Egypt. Using specific IgG-ELISA kits, we screened sera collected from humans and camels in the two survey areas, Sohag and Asyut. Related data were recorded through in-person interviews with human participants and camel owners before subsequent analysis based on the serology data.

In the present study, a disease seroprevalence equal to 12.09% was revealed in humans. An extensive literature search using different tools revealed that there is a paucity of recent epidemiological data on the prevalence and risk factors of DF in Egypt. This gap in literature has existed for over 45 years as no updates have been published since Darwish *et al*. [18] reported a DF prevalence of 0.3% among university students [18]. Notably, our result is comparable with recent survey reports from the Middle East and African countries. For instance, in Sudan, prevalences of 27.7%, 9.4%, and 47.6% of surveyed humans were documented to have DENV IgG antibodies in 2011, 2013, and 2018 [19-21], respectively. A prevalence of 6% and 15.7% was previously reported in two published Nigerian and Kenyan human studies [22,23], respectively. Moreover, in studies from Saudi Arabia, a range of 0.1-48.75% was documented [24-26]. However, 5% and 7.6% infection rates in humans were stated in Iran in 2013 and 2014 [27,28], respectively.

The impact of different variables on the disease prevalence was evaluated. Fisher’s exact test and OR were used to analyze the impact of each risk factor on the DF prevalence individually as mentioned earlier in the methodology section.

In humans, DF seropositivity did not differ significantly by any of the analyzed factors; however, variations were found after data were computed by the OR. Rural residents had higher odds of exposure to DF than urban and this could be explained by the availability of mosquito breeding habitats, such as water barrels, discarded plastic containers, and discarded tires, in the rural rather than urban residential areas of Upper Egypt. These kinds of containers were documented as preferred breeding habitats for the DENV mosquito in a number of published studies in Africa [29-31]. Accordingly, measures to eradicate the mosquito breeding habitats should be adopted.

In this study, males had a higher prevalence than females which is in agreement with Eldigail *et al*. [21] and Koh *et al*. [32], but in contradiction to the observation of Nava-Aguilera *et al*. [33]. A possible reason that males are more at risk of infection compared to femalesis the propensity for males to have higher exposure rates to mosquitoes, given that males are often subject to more outdoor activities.

In the present investigation, the disease prevalence was found to be inversely proportional to human age. For instance, humans of <21 years were at the highest risk followed by those between 21 and 40 years. Our finding is consistent with the previous studies indicating that the disease is more prevalent in younger ages [21,32,33]. Despite these consistencies regarding age and DF risk between our observations and other reports, no conclusions can be drawn from the present finding due to the small sample size of this age group (<21 years, n=8).

This study indicated that exposure to mosquitoes played a remarkable role in increasing the risk of getting DF infection. This exposure is likely to occur during the day when *Aedes* mosquitos are actively feeding. For the same reason, as reported in a number of studies, the use of mosquito nets at night was found to be ineffective in DF prevention [21,34]. Indeed, the majority of people who agreed to participate in this study reported the use of mosquito nets during the night. While this practice is necessary to protect against insect bites in the evening hours, it is still insufficient in conferring protection against exposure to mosquitoes like *Aedes* which are active during the day. Thus, the use of mosquito nets in the day and night combined with the application of other mosquito control measures is highly recommended.

In this study, 81.81% of the humans which were seropositive for DENV experienced breakbone fever while 18.19% did not. While the presence of breakbone fever can be predictive for a history of the disease, the fact that the ELISA assay utilized here measures IgG which remains detectable for extended periods (up to several months) [35], makes a precise determination of infection timing quite difficult.

As mentioned earlier, humans are considered the primary reservoirs for DENV though the disease can also affect non-human primates. However, little is known about the role of other animals as reservoirs for DENV. Some evidence exists for natural infection of domestic dogs with DENV [36]. That report is alarming since it confirmed the circulation of the virus among one common companion animal which lives in close proximity with humans. Camels can carry infectious diseases with no apparent symptoms as noted with several viral zoonoses, including foot and mouth disease, Rift Valley fever, and MERS coronavirus [17,37]. Based on this precedence, we hypothesized that camels could contract the DENV infection and remain asymptomatic. Within Egyptian culture, there is a growing demand for camel meat for human consumption and currently, local production of this animal is exceeded by demand. Thus, Egypt depends heavily on the importation of these animals to meet the growing demand [38]. In this study, we documented the presence of specific antibodies against DENV in 3 of the 91 camels examined (3.3%). Interestingly, all the seropositive camels reported in this study experienced fever and decreased appetite within the 14 days before sample collection. Because this finding is alarming as it suggests that camels could be naturally infected by DENV and therefore provides an additional environmental niche for the virus, it should be treated cautiously until further confirmed. In particular, the role of camels as maintenance or accidental hosts needs to be clarified.

Among the computed sub-variables, females, imported, and work animals were at increased risk of getting the infection compared to males, local, and food animals, respectively. Of the 15 female camels examined in this study, only one animal reacted positively to the IgG antibodies and it is worthy to note that this animal experienced fever and abortion shortly before sampling.

Camel breed did not significantly affect the disease prevalence; however, imported animals were at 3 times greater risk compared to local breed animals. Before being released and distributed to various areas nationwide, camels imported to Egypt from Sudan typically remain in quarantine for 10-15 days in Abu Simbel or Shelateen facilities in Aswan, Egypt [39]. This shipping stress could represent a potential risk factor for the acquisition of dengue infection in imported animals. However, the use of the IgG ELISA rather than IgM ELISA in this study cannot offer insight regarding the timing of the initial exposure to the virus. Therefore, determining if a positive correlation exists between travel-related stress and DENV infection will be difficult to track among imported animals.

## Conclusion

This survey reports the circulation of DENV-specific IgG antibodies among humans and camels in Upper Egypt in areas geographically distinct from the two previous outbreak areas, Dairot and El Quseir. Although this preliminary information is an important indication that the virus has likely expanded its geographical spread and hosts, a more comprehensive study, including larger sample size, more survey regions, and more advanced molecular techniques, is required. Based on our findings, we recommend health education of the public on the danger of the disease, management of mosquito habitats, and application of anti-mosquito control measures are needed to prevent increased DENV incidence in Upper Egypt.

## Authors’ Contributions

This manuscript was drafted from the Master thesis research of MOH under the supervision of ASMS and MFNA. ASMS and MFNA designed and coordinated the study. ASMS and MOH shared in sampling and ELISA work. MFNA contributed to literature collection, data analysis, and writing of the manuscript. ASMS edited and reviewed the manuscript. All authors read and approved the final manuscript.
